# Bengali-Sign: A Machine Learning-Based Bengali Sign Language Interpretation for Deaf and Non-Verbal People

**DOI:** 10.3390/s24165351

**Published:** 2024-08-19

**Authors:** Md. Johir Raihan, Mainul Islam Labib, Abdullah Al Jaid Jim, Jun Jiat Tiang, Uzzal Biswas, Abdullah-Al Nahid

**Affiliations:** 1 Electronics and Communication Engineering Discipline, Khulna University, Khulna 9208, Bangladesh; mj.raihan2@gmail.com (M.J.R.); mainulislamlabib@gmail.com (M.I.L.); jaidjim@gmail.com (A.A.J.J.); uzzal.biswas@ece.ku.ac.bd (U.B.); 2 Department of Electrical and Electronics Engineering, Trust University, Barishal 8200, Bangladesh; 3 Centre For Wireless Technology (CWT), Faculty of Engineering, Multimedia University, Cyberjaya 63100, Malaysia

**Keywords:** Bengali sign language (BdSL), squeeze excitation (SE), convolutional neural network (CNN), SHAP

## Abstract

Sign language is undoubtedly a common way of communication among deaf and non-verbal people. But it is not common among hearing people to use sign language to express feelings or share information in everyday life. Therefore, a significant communication gap exists between deaf and hearing individuals, despite both groups experiencing similar emotions and sentiments. In this paper, we developed a convolutional neural network–squeeze excitation network to predict the sign language signs and developed a smartphone application to provide access to the ML model to use it. The SE block provides attention to the channel of the image, thus improving the performance of the model. On the other hand, the smartphone application brings the ML model close to people so that everyone can benefit from it. In addition, we used the Shapley additive explanation to interpret the black box nature of the ML model and understand the models working from within. Using our ML model, we achieved an accuracy of 99.86% on the KU-BdSL dataset. The SHAP analysis shows that the model primarily relies on hand-related visual cues to predict sign language signs, aligning with human communication patterns.

## 1. Introduction

For people who are deaf or have hearing impairments, hand sign language, also known as sign language, is an essential form of communication. In Bangladesh, where an estimated 2.5 million people suffer from hearing loss, sign language is essential for bridging the communication gap and promoting social inclusion [1]. There are several reasons why sign language is significant. First of all, it ensures that deaf people can communicate effectively and have equal access to information. They may find it difficult to communicate their needs, comprehend instructions, or have conversations if they do not know sign language. Sign language enables the Deaf community to communicate effectively by using signs, gestures, and facial expressions, improving their social interactions and quality of life. Additionally, sign language helps deaf children learn. Sign language can be used as a teaching tool to help deaf people in Bangladesh, where their literacy rate is significantly lower than the general population’s. This will help them increase their knowledge and skills. Sign language enables deaf students to interact with their peers and teachers, take part in class discussions, and have access to educational resources. They are equipped to overcome obstacles and realize their full potential thanks to this inclusive education. Statistics highlight sign language’s significance in Bangladesh even more. Over 90% of deaf children worldwide are born to hearing parents, who may at first find it difficult to effectively communicate with their child, according to the World Health Organization (WHO) [1].

Machine learning (ML) research for sign language sign detection is a rapidly developing area with enormous potential. Researchers have been working on systems that can precisely interpret and translate sign language signs into text or spoken language using computer vision techniques and deep learning algorithms [2,3,4,5]. By enabling real-time communication between sign language users and non-signers, these systems can advance accessibility and inclusivity. Research is currently being conducted to enhance the classification and recognition of sign language signs, investigate new datasets, and create reliable models that generalize well across various sign languages [6,7,8,9,10,11]. Exciting changes exist for ML to improve communication and give the Deaf community more power. The earliest application of ML in sign language recognition came from Pugeault et al. who used image depths and hand positions as discriminative features where the “Random Forest” (RF) model was used as a classifier [6]. For the appearance and depth of image collection, the system uses a Microsoft Kinect device. For hand detection and tracking, it uses the OpenNI+NITE framework. When neural networks underwent great development, novel feature extraction methods started being developed to use the new firepower behind deep learning. Extraction techniques such as (a) histogram technique, (b) Hough, (c) OTSU’s segmentation algorithm, and (d) segmentation and extraction with edge detection were used by Kulkani et al. to differentiate between different hand signs [7]. Due to the fact that the system dealt with pictures of bare hands, the user was able to interact with it naturally. A feature vector of an image after processing and conversion was compared with the feature vectors of a training set of signs. The system is more flexible because it allows for the translation, scaling, and rotation of the sign within the image. As time passed, the need for a reliable sign language detection system compelled researchers to create reliable systems.

One of the first suggested systems was the mechanism developed by Rahaman et al., which determined the likely hand sign from the captured image [8]. The system used feature-based cascaded classifiers with Haar-like properties to determine the hand sign in each frame. The system extracted the hand sign from the detected hand area based on the hue and saturation values that correspond to the color of human skin. The binary images were then categorized using the K-nearest neighbors (KNN) classifier by contrasting them with binary images of hand signs that had previously undergone training. With modern deep learning technologies, Shanta et al. used SIFT transformation with a convolutional neural network (CNN) to classify the hand sign images. They also showed that using the SIFT feature increases the accuracy of the CNN when detecting Bangla sign language [9]. With the advent of generative AI, Shishir et al. developed EsharaGAN, a model for creating Bangla sign digits based on InfoGAN, or information maximizing generative adversarial networks [10]. The IsharaLipi dataset was used to train this model, which used a 13-layer network architecture with input, dense, convolutional, transpose, activation, and batch normalization layers. This model minimized loss function and computation power while producing images that were not distorted and faithfully reproduced reality. Tanh and ReLU were employed as the activation function. Given that this generative model has an impressive inception score of 8.77, it produced an exceptional result.

Similarly, Rafi et al. used a pre-trained MobileNetV2 model along with conditional GAN to develop a lightweight model which can be used on modular devices to deliver one-stop services in Bengali sign recognition [12]. Using the discriminatory power of deep transfer learning, Das et al. suggested a hybrid model for the automatic recognition of Bangla sign language (numerals and alphabets) that combines a deep transfer learning-based convolutional neural network with a random forest classifier. On the “Ishara-Bochon” and “Ishara-Lipi” datasets, the presented system’s overall performance was confirmed [11]. The first comprehensive multipurpose open access datasets for Bangla sign language (BSL) were the “Ishara-Bochon” and “Ishara-Lipi” datasets of isolated numerals and alphabets, respectively. To design better modular systems for Bengali sign language detection, Ahammed et al. proposed a lightweight model with comparably better performance with augmented information from angular transformation for personal uses. They developed a mobile application for both Android and iOS devices, which has helped numerous people in communicating with their loved ones [13].

The Deaf community in Bangladesh heavily relies on sign language. It improves communication, encourages inclusive education, strengthens parent–child bonds, and makes essential services more easily accessible. Recognizing the value of sign language and making investments in its promotion and education will help to create a more inclusive society where everyone’s rights and needs, regardless of hearing ability, are respected and met. An automatic Bangla sign language (BSL) detection system using deep learning on a Jetson Nano edge device was developed by S. Siddique et al. [14]. The system was trained on the Okkhornama database and a custom dataset of 49 categories with 3760 images. It achieves high detection accuracy, with the Detectron2 model performing best with a mAP@.5 of 94.915. The system’s contribution lies in providing a simple and affordable solution for real-time BSL detection. However, it has not yet been implemented on smartphones, limiting its accessibility to a broader user base. Not everyone has access to a Jetson Nano, and using such a device can be inconvenient for everyday use, making a smartphone-based solution more practical and widely available. A study performed by S. Renjith et al. reviews 95 AI-based research papers on sign language recognition, focusing on language categorization, sign type, signing modes, processing techniques, classification methods, and evaluation measures [15]. Extensive studies were found in Chinese, Arabic, and American sign languages, with SVM and CNN showing high performance in machine learning and deep learning, respectively. However, the review does not address the development of smartphone-based applications or include research on explainable AI methods, limiting practical accessibility and understanding of the underlying models.

In this research, we made significant contributions to bridging the communication gap between deaf and hearing individuals through the development of an advanced sign language recognition system. We created a CNN with squeeze excitation (SE) network, achieving an impressive 99.86% accuracy on the KU-BdSL dataset. This high level of accuracy is crucial for reliable real-world applications. Our integration of the SE block enhances the model’s performance by focusing attention on important channel features in the images. To make this technology accessible, we developed a smartphone application, bringing the power of our machine learning model directly into users’ hands. This practical implementation is vital for widespread adoption and real-world impact. Furthermore, our use of SHapley Additive eXplanation (SHAP) provides valuable insights into the model’s decision-making process, revealing that it primarily relies on hand-related visual cues, similar to human communication patterns. This transparency in AI decision-making is essential for building trust and understanding in the technology. Together, these contributions represent a significant step forward in automated sign language recognition, potentially revolutionizing communication between Deaf and hearing communities in everyday life.

## 2. Methodology

**Figure 1:**
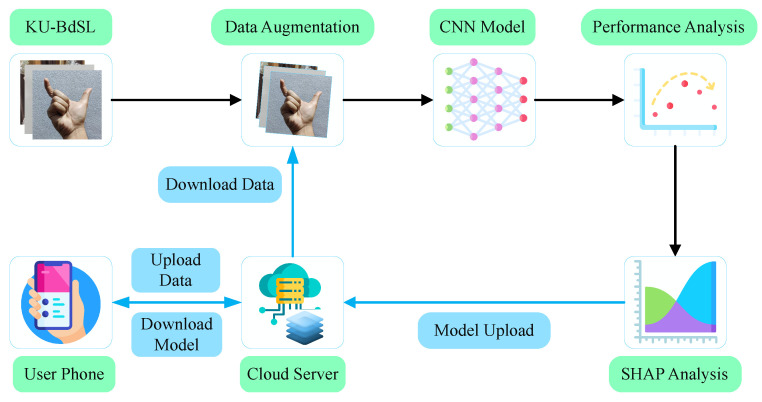
Workflow of the proposed sign language prediction framework.

The prediction of sign language signs falls under the supervise learning (SL) problem as the data we used were labeled. Figure 1 shows the workflow of the proposed framework. The KU-BdSL contains around 1500 samples [16,17]. We applied several data-augmentation techniques to make more samples so that the model became more robust. Then, this set was given to a CNN with an SE block to further increase the model’s performance by focusing attention to the image channels. To further investigate the model’s performance, we used the SHAP analysis. The SHAP analysis helps us to understand the influence of the features on the models when predicting a certain sample. This step helps to understand which part of the images is influencing the model to predict the class. This model is then distributed to the user’s handset, which can be used locally by the user to predict SL signs using the phone’s camera or stored images. Additionally, the user can provide labeled data through the application which are stored in the cloud. Again, these users’ data are used to further improve the model’s performance on real-world data. The collection of large amounts of data can also open a new door to other types of gesture recognition beyond the prediction of SL signs.

In the latter sections, we discuss the KU-BdSL dataset in great detail. Also, we provide a foundational background on the CNN–SE network. Later, we discuss how the model is integrated with smartphones and creates other features like collecting data and prediction from cameras.

### 2.1. Data Preproces

**Figure 2:**
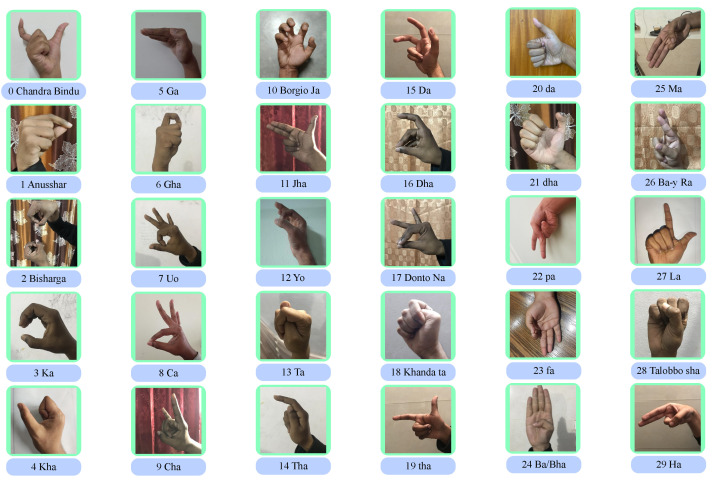
Thirty unique samples from the KU-BdSL dataset.

We collected the publicly available KU-BdSL dataset from [16,17]. The dataset contains 1500 samples of 30 Bengali alphabets captured from 33 participants among which 25 are males and 8 are females. The images contain 512 × 512 pixels with 8-bit Red, Green, and Blue (RGB) channels. The dataset is rather robust as the dataset was collected using several smartphone cameras and various light conditions. Figure 2 shows all thirty samples from the KU-BdSL dataset. Also, the dataset is balanced as each type of class has the same number (50) of samples.

**Figure 3:**
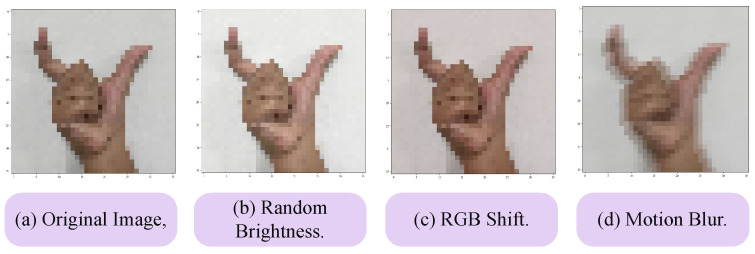
Data augmentation process of a random sample from the KU-BdSL dataset.

However, to make the dataset more robust, we performed several data-augmentation techniques. The augmentations of a sample image are shown in Figure 3. We converted the samples into 36 by 36 size and performed random brightness, RGB shift, and motion blur. The parameter values of these augmentation techniques are given in Table 1. We considered these augmentations as they may arise when users will be predicting the SL using their smartphone. After this process, we finally created a subset containing 15,000 samples. We then split the subset in 70/30 format which gave us 10,500 training samples and 4500 testing samples.

### 2.2. CNN-SE

The CNN was first introduced by Yen LeCun et al. in 1980, for handwritten digit recognition [18,19]. Since then, it has been used in various computer vision-related problems. One of the remarkable features of the CNN is the affine transformation of the image as it feed-forwards through the network. This allows the model to recognize the feature that shifted, slightly tilted, or wrapped within the image. The idea of local receptive fields, in which each neuron in a layer is coupled to just a small part of the input, is utilized by CNNs. This enables the network to pay attention to regional patterns and visual properties. Additionally, CNNs use shared weights, which means that the same set of weights is applied throughout the entire image to each local receptive field.

In CNNs, multiple kernels slide over the image and perform an operation called convolution (Equation (1)). The convolution involves the element-wise multiplication of kernel weight value with the input pixel value in a small region of the input image. The result of this convolution yields a feature map that may contain important features that can be useful for the model. A number of these kernels can be used to capture a large number of feature maps to improve the model’s prediction ability. Then, an activation function is used to introduce the non-linearity to the model so that the model can learn more complex relationships. One of the most commonly used activation functions is called ReLu (Equation (2)). Another layer used in the CNN is the pooling layer that reduces the size spatial dimension while retaining the important features of the feature map. The pooling layer can use the average pooling method or max pooling depending on which performs the better. In the final layer of the network, we used the SoftMax activation function (Equation (4)), which gives the probability of the sample being in one of the 30 classes.

(1) $$\mathrm{Convolution},z^{l}=h^{l-1}\times W^{l}$$

(2) $$\mathrm{ReLU}=\max(0,z_{i})$$

(3) $$\mathrm{Fully}\text{-}\mathrm{Connected}\;\mathrm{layer},z_{l}=W_{l}\times h_{l-1}$$

(4) $$\mathrm{Softmax}=\frac{e^{z_{i}}}{\sum_{j}e^{z_{j}}}$$

**Figure 4:**
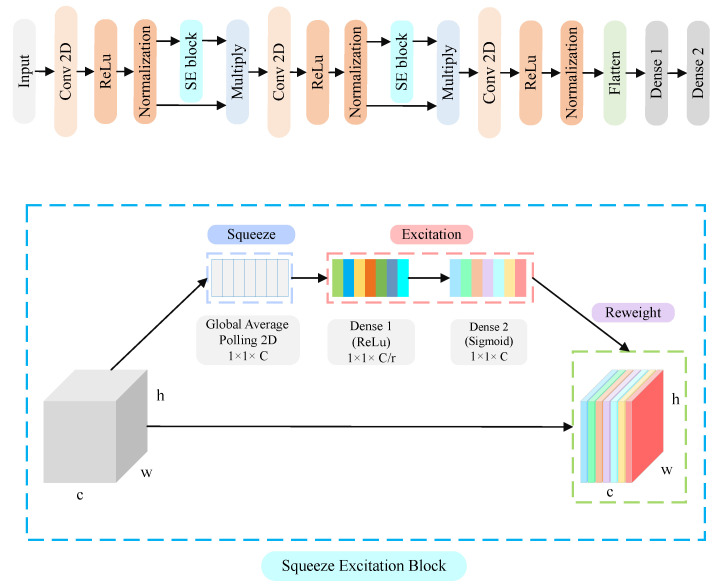
CNN architecture with SE block.

We also improved the representational power of the CNN using the SE network. The SE was originally proposed by J. Hu et al. in 2018 [20]. The first part of the SE network called “squeeze” applies global pooling to reduce the spatial dimension and preserve the channel dimension. The second part, called the excitation part, uses the dense layer and activation layer to produce a channel-wise weight representing the importance of that channel. The complete diagram of the architecture is given in Figure 4. The formula of the squeeze part $F_{sq}\left(u_{c}\right)$ is given by

(5) $$z_{c}=F_{sq}\left(u_{c}\right)=\frac{1}{H\times W}\sum_{i=1}^{H}\sum_{j=1}^{W}u_{c}\left(i,j\right)$$

where the height H and width W of the input image $u_{c},c$ is the channel. The excitation part is given by

(6) $$s=F_{\mathrm{ex}}\left(z,W\right)=\sigma \left(W_{2}\mathrm{ReLU}\left(W_{1}z\right)\right)$$

where weight $W_{1}\in R^{\left(Cc/r\right)}$ and weight $W_{2}\in R^{\left(Cc/r\right)}$, and *r* is the reduction ratio. The larger the value of r, the smaller the intermediate representation which we fixed at 6 and 9. In the three convolutional layers, we used filter sizes 27, 28, and 35. We used the “categorical cross entropy” as the loss function and the learning rate was set to 0.0001. We used two fully connected layers (Equation (5)) having 128 and 64 nodes followed by an output layer with 30 nodes and the softmax function to output the sign class probability.

### 2.3. Performance Metrics

To measure the performance of the ML model, we used accuracy, precision, recall, and F1-score. The general correctness of a model’s predictions is measured by accuracy. It determines the proportion of correctly identified examples in all of the dataset’s instances. Although accuracy is a valuable indicator, it can be deceiving in datasets with skewed distributions of classes. The capacity of a model to accurately distinguish positive cases from all instances it predicted as positive is referred to as precision, while recall indicates how well the model can distinguish between positive examples from all of the real positive cases in the dataset. A model’s performance may be evaluated fairly using the F1-score, a single statistic that combines precision and recall. It is the harmonic mean of precision and recall. The formulas for calculating the accuracy, precision, recall, and F1-score are given in Equations (7), (8), (9), and (10), respectively.

(7) $$\mathrm{Recall}=\frac{TP}{TP+FN}$$

(8) $$\mathrm{Precision}=\frac{TP}{TP+FP}$$

(9) $$\mathrm{Accuracy}=\frac{TP+TN}{TP+TN+FP+FN}$$

(10) $$F1\text{-}\mathrm{Score}=2\cdot \frac{\mathrm{Precision}\cdot \mathrm{Recall}}{\mathrm{Precision}+\mathrm{Recall}}$$

The formulas represent key evaluation metrics for classification models. Recall measures the ability to identify all relevant instances (true positives out of actual positives). Precision gauges the accuracy of positive predictions (true positives out of predicted positives). Accuracy reflects the overall correctness of predictions (correct predictions out of all predictions). The F1-score harmonizes precision and recall, providing a single metric that balances both. Here, TP (True Positives) refers to correctly predicted positive instances, TN (True Negatives) to correctly predicted negative instances, FP (False Positives) to incorrectly predicted positive instances, and FN (False Negatives) to incorrectly predicted negative instances.

In addition, we used SHAP to better interpret the ML model. SHAP was first introduced by S. Lundberg et al. in 2017 [21]. It helps us comprehend the significance and effects of various input variables by offering insights into the contribution of each feature to a model’s output. SHAP’s fundamental idea is based on Shapley values from cooperative game theory, which determine each feature’s average contribution to overall potential combinations. In sign language, SHAP can be used to identify how the model is using the feature to predict a certain class so that we can better improve the model. Also, it helps us to explore the black-box nature of the model, which leads to transparency and trust.

### 2.4. Smart Application Development

One of the best ways to reach the developed model to users is through a smartphone. In today’s world, a smartphone is an essential thing and it can be found on anyone. Thus, developing an Android application ensures that the model reaches the masses and can be used as a tool to improve people’s communication gap. With a sign language ML model available on smartphones, deaf and non-verbal people may communicate on their own. In a variety of contexts, including employment, social events, and when asking for help in public areas, they can utilize their smartphones as a communication tool. Their confidence and social engagement may increase as a result of their independence.

**Figure 5:**
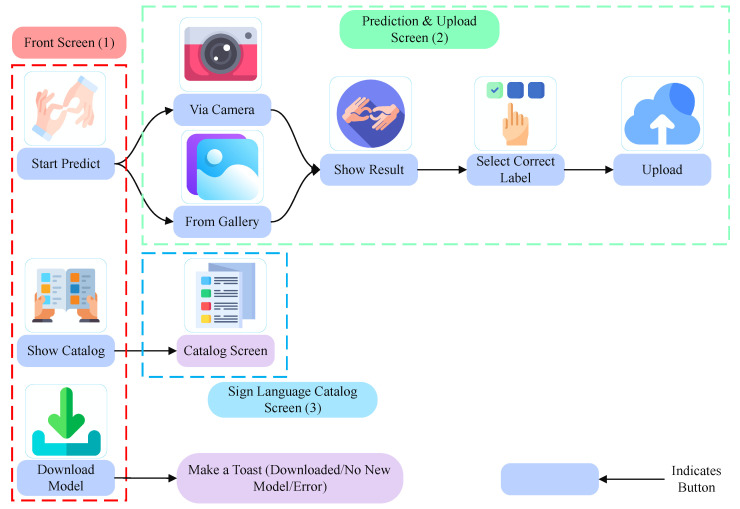
All the functionalities of the proposed smartphone application for sign language prediction.

We chose to develop a very simple yet very effective application with only essential features. Thus, it will be very straightforward and useful for people of all ages. Figure 5 shows all the available functionalities of the smartphone application. The application offers several features such as a user-friendly catalog, cloud-based model updates, offline sign language prediction, and uploading user data.

The catalog shows which sign indicates which Bengali alphabet item. This will help those people who do not have any knowledge about the Bengali sign language alphabet. Thus, they can easily navigate through different categories and access a wide range of signs for everyday communication. When predicting the sign, the user can pick the images from the gallery or directly from the camera. If the model predicts the wrong sign, the user can select the correct sign and upload the data along with the label to the server. This process will ensure the robustness of the model as it can be trained on a variety of samples. The ML model can be generalized on a larger set of data. On the cloud side, we automated the process of collecting the data from the server and training on the new dataset on its own. This step will reduce the human intervention to train the model.

We used modern technologies to build the application so that it can be coded once but run on many devices. Thus, we used the Flutter framework by Google to create the main functionality of the application. The application created by Flutter can be run on Android, IOS, MacOS, Linux, and Windows. However, for simplicity, we only configured it to run on Android devices for now. The application will come with an inbuilt ML model which can be updated to the latest version anytime by the user and show the availability of the new model on the server side. The server will only hold the data uploaded by the user and the latest model. We used the freely available Google Firebase to both store the user’s uploaded data and the latest model. We will perform the training of the model on a local machine as this will reduce the cost of hosting a server. On the local machine, a Python script will automatically download the new user data from the Firebase cloud and continue to train the model. After training the model, it will upload the latest model to the cloud. The script can also be automated to run once a month. Thus, a new model will be available once a month for the user to download.

## 3. Results

In this section, we present the results we obtained during our research. In the first subsection, we show the results of model training with the CNN with SE block. Then, we interpret the model using SHAP and show how it can be used to understand how the model works. Finally, we demonstrate the actual smartphone application which can be used by the end-user anytime and anywhere in the world.

### 3.1. Results and SHAP Analysis

**Figure 6:**
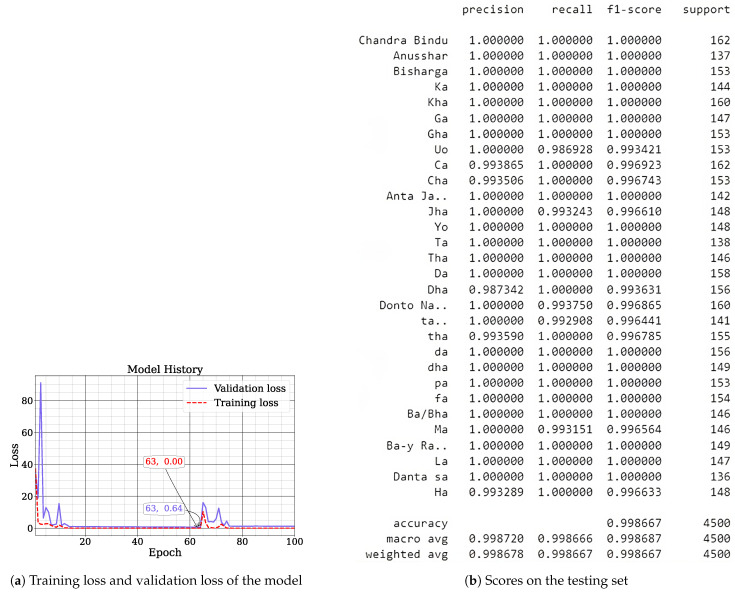
Results on the testing set.

**Figure 7:**
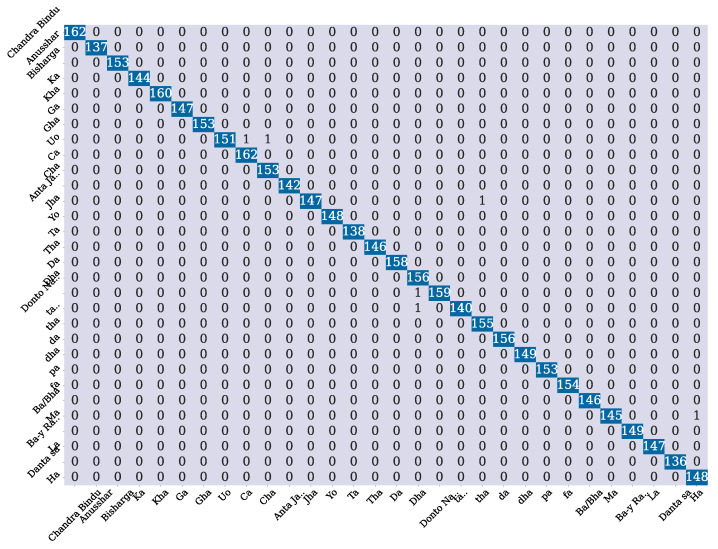
Confusion matrix of the model on the test set.

As we stated before, we used a total of 15,000 samples using several augmentation techniques. We used 70% of it as a training set and 30% for a testing set. Figure 6a shows the training loss and validation loss at each epoch. Along the x-axis, we have epoch, and along the y-axis, we have the cross-entropy loss. From the graph, we find that the model is converging very quickly as the training and validation loss is decreasing at a significant rate. This can also be due to the fact of using the SE block which may help the model to prioritize the channel, thus resulting in quick convergence. The results of the testing set are shown in Figure 6b. In Figure 7, we show the confusion matrix of the model, which indicates that the model is performing very good on the testing set.

On the testing set, we achieved an accuracy of 99.86%. On this same dataset, G. S. Surjo et al. explored VGG16, ResNet50, and MobileNetV2, achieving up to 98% accuracy, as shown in Table 2 [22]. However, our method surpasses this with 99.86% accuracy. N. Begum et al. utilized Xception architecture with quantization and layer compression, reaching a 99% F1-score [23]. A. S. M. Miah et al. employed a CNN with data augmentation techniques, attaining 99.6% accuracy [5]. The integration of the SE network allows for adaptive feature recalibration, while SHAP analysis provides interpretability elements missing from previous approaches. This combination not only achieves higher accuracy but also offers insights into the model’s decision-making process, making our method more robust and explainable for Bengali sign language recognition tasks.

**Figure 8:**
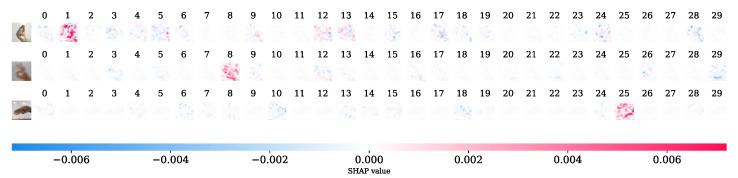
Interpreting the CNN model using SHAP.

In addition, we performed the SHAP analysis on the model. Figure 8 shows the model’s interpretation of several images picked randomly. The presence of the red color influences the model into predicting the class it is predicting while the blue color region reduces the model’s prediction of that class. The labels of these images are given in Figure 2. As we can see, the model has accurately classified each of the signs. For example, if we take the first test image, it is the sign for “Anusshar”. From the figure, it can be seen that there are more red dots on the second class (labeled as “1” from the left side); thus, the model predicts it as Anusshar. A similar observation can be seen in the other samples. The model predicted all these samples accurately.

### 3.2. Smartphone Application (KU-BdSL)

**Figure 9:**
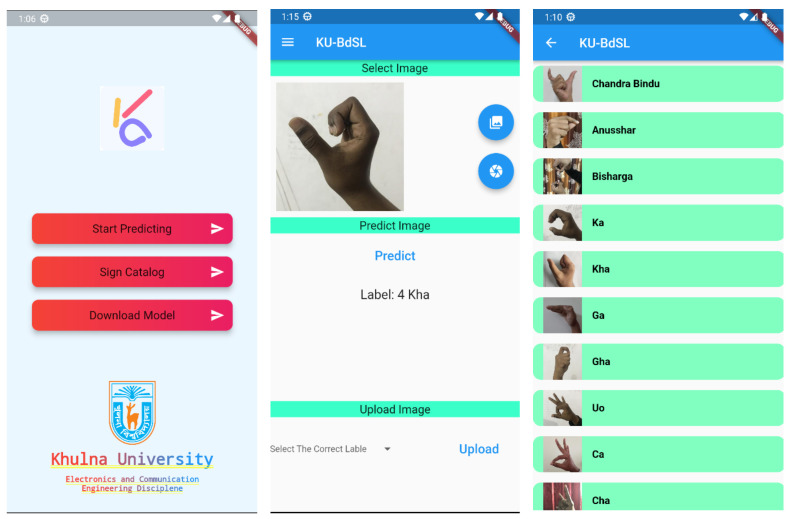
Developed smartphone application.

In this section, we present the smartphone application that we discussed in the Methodology Section. On the front screen, the application shows three main buttons, as shown in Figure 9. The first button takes the user to a different screen where the user can predict and upload the data to the cloud. The second button takes the user to another screen where the user can view the catalog of the available signs the model can predict. The third button simply downloads the latest version of the ML model from the Firebase server depending on the availability. Also, we created a sidebar which the user can use to quickly jump to these screens. On the prediction page, the user can use the gallery button to pick any image from the gallery and predict its class. The user can also click on the camera button to capture the image in real time and predict the class. The user can upload the sample by selecting the correct label from the drop-down selection menu and clicking on the upload button.

Now, when the user clicks on the catalog page, it simply shows a picture of a sign and a text of what the sign means. This will help non-signers to educate themselves about sign language. Also, they can simultaneously teach themselves whether their hand gesture is correct enough to make the sign they are trying to express. They can do this using the model predicting ability to predict their hand sign and find whether the model is predicting the correct sign they are giving. If the model predicts another sign than the one they are trying to make, the user can try a better way to show their hand sign. In this way, the application can also be used to teach people who do not know sign language.

**Figure 10:**
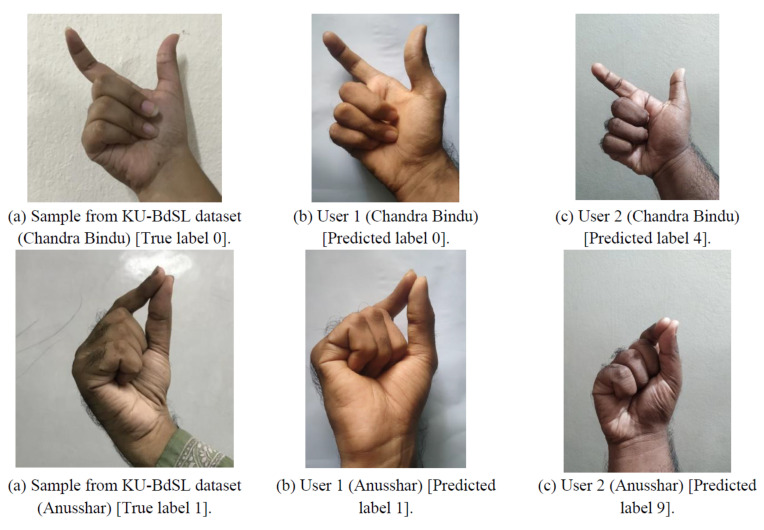
Predicated sign of two users.

**Figure 11:**
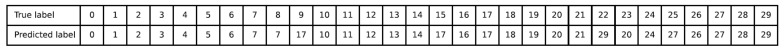
Predicted outcome of all the samples of User 1.

**Figure 12:**
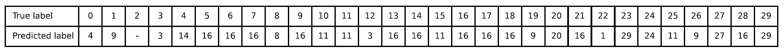
Predicted outcome of all the samples of User 2.

Additionally, we conducted an evaluation of the hand sign prediction application with two consenting participants. As illustrated in Figure 10, the model successfully identified the “Chandra Bindu” and “Anussar” signs for User 1 with a high degree of accuracy. However, the model exhibited difficulties in correctly predicting these signs for User 2. A comprehensive analysis of the predictions across all samples is presented in Figure 11 and Figure 12. These figures provide a comparative overview, highlighting that the model accurately identified 24 out of 30 signs for User 1, corresponding to an 80% success rate. In contrast, the model correctly predicted only 8 out of 30 signs for User 2, yielding a significantly lower accuracy of approximately 26.7%. These results suggest a potential variability in model performance based on individual user differences.

The discrepancy between the model’s accuracy on test data (99.86%) and its performance in real-world scenarios (80% accuracy for User 1, as shown in Figure 11) can be attributed to several technical factors. Firstly, the test dataset (KU-BdSL) may be more uniform and less varied than real-world inputs, leading to overfitting wherein the model performs exceptionally well on the images available in the original dataset but struggles with unseen variations in lighting, hand orientation, or individual differences in hand shape and skin tone. Secondly, the limited number of samples in the KU-BdSL dataset may not capture the full range of real-world variability, leading to a model that is less robust when deployed in practice. To address this issue, we employed several augmentation techniques, as discussed in the “Data Processing” subsection, to enhance model robustness. However, substantial amounts of additional data are necessary to further improve the model’s overall performance, which can be collected through the application. Lastly, the smartphone application’s image capture process could introduce variability (such as resolution differences or slight motion blur), further contributing to the observed performance drop. These factors highlight the challenges of transitioning from controlled environments to real-world applications.

## 4. Discussion

In this section, we discuss how the framework can benefit the technical side of ML and real-world humans. Also, the framework raises questions about privacy, security, and future improvements. First, the prospered model is based on a very simple CNN and SE block. The model tries to recognize the pattern with the given image and predict a certain class. Also, to improve the performance, channel-wise attention was given to implement the SE block. Both of these techniques are well-known in the ML field and are widely used in image recognition. Our paper focuses on using these modern technologies to solve a very simple yet very impactful problem of sign language prediction to better human life. We have shown that the ML technique can be used to predict Bengali sign language very efficiently. Furthermore, we used the SHAP analysis to break down what the model sees in a given image. This can help the scientific community to better understand how the model works to predict sign language.

The developed smartphone application certainly brings the prediction capability of the ML model to the end user. The catalog pages help people to learn about sign language and provide them with a way to practice on their own using the model’s prediction capability. Certainly, this will help people to learn a new language and reduce the communication gap between deaf and hearing people. The users can donate anonymous data with labels, which can be used to build a larger sign language dataset. It will be beneficial to the scientific community to use larger real-world data from a variety of samples to create a more efficient model and smartphone application.

Our CNN–SE model’s impressive 99.86% accuracy on the KU-BdSL dataset demonstrates the effectiveness of our approach in accurately recognizing sign language gestures. Our use of SHAP analysis provided valuable insights into the model’s decision-making process, confirming that it primarily relies on hand-related visual cues, aligning with human communication patterns. This transparency not only aids in understanding the model’s functionality but also builds trust in the technology. Furthermore, the app’s features, such as the catalog pages for learning sign language and the ability for users to contribute anonymous data, demonstrate the practical implementation and potential for continuous improvement of our system. These results collectively showcase how our research has made significant strides in bridging the communication gap between Deaf and hearing communities.

The proposal also raises questions about security as it deals with public data. We have developed the server side using Google Firebase technology, which provides its layer of security. Furthermore, the implementation of the model is performed on a local machine on the data collected from the server once every month and clears out the storage. However, we know no system is secure; thus, measures should be taken at every step to ensure the security of the user data. Also, the user may provide wrong-labeled data intentionally or internally. Hence, we can implement another layer of security to verify the given images before they are used to train the model. Otherwise, its performance may be reduced and it may provide wrong results.

In future work, there are many ways to improve the current framework. We can collect a larger amount of data and use them to train the model. We can implement a better CNN, such as Transformers, to improve the model’s capability of recognizing patterns. We can also incorporate transfer learning which may further improve the model’s performance. We can take real-world user feedback to introduce new features into the smartphone application. We can also allow the user to introduce their personal sign or gesture, usable by them only, to be able to communicate more rapidly.We can also add multilingual support or translation so that the application can be used by people of different languages, breaking the barrier of language difference. In short, there are many ways to improve the current proposal and make a better application to aid humans.

## 5. Conclusions

In conclusion, our framework significantly advances communication between deaf and hearing individuals through sign language. By integrating a CNN enhanced with an SE block and utilizing SHAP analysis, we improved performance and gained deeper insight into feature influence, achieving an impressive accuracy of 99.86% on the testing set. The SE networks notably augment the CNN’s representational capabilities. To ensure practical application, we developed a user-friendly mobile app using Flutter, supporting real-time and offline sign language prediction. Users can contribute labeled sign data to continually improve the model. Efficiency is maintained by handling uploaded images and models on a server, with automated monthly updates incorporating new data. Real-world testing on smartphones indicates the framework’s potential to significantly aid in daily communication, underscoring its practical applicability and transformative impact in bridging communication gaps.

## Figures and Tables

**Table 1:** Parameters of the augmentation technique.

Augmentation Technique	Parameter Name	Values
Random Brightness	probability	0.7
RGB Shift	R shift limit	5
	G shift limit	5
	B shift limit	5
	probability	0.7
Motion Blur	Blur limit	7
	probability	0.7

**Table 2:** Performance comparison between the proposed model and other architectures on the KU-BdSL dataset. (The highest achieved performance is shown in bold.)

Research Conducted by	Methodology	Metric	Score
Surjo et al. [22]	VGG16	Accuracy	98%
Surjo et al. [22]	ResNet50	Accuracy	97%
Surjo et al. [22]	MobileNetV2	Accuracy	95%
Begum et al. [23]	Xception architecture + Quantization + Layer Compression	F1-Score	99%
Miah et al. [5]	CNN and data augmentation techniques	Accuracy	99.6%
Our	CNN + SE Network + SHAP Analysis	Accuracy	**99.86%**

## Data Availability

The dataset we have used in our study is publicly available on the Mendeley website: https://data.mendeley.com/datasets/scpvm2nbkm/4, accessed on 5 November 2023.

## Abbreviations

The following abbreviations are used in this manuscript:

CV	Computer Vision
ML	Machine Learning
CNN	Convolutional Neural Network
SE	Squeeze Excitation
SHAP	SHapley Additive eXplanation
KU-BdSL	Khulna University Bengali Sign Language dataset
WHO	World Health Organization
KNN	K-Nearest Neighbors
RGB	Red, Green, and Blue
